# Supplementary material for: CAJAL enables analysis and integration of single-cell morphological data using metric geometry
Source: Nat Commun. 2023 Jun 21;14:3672. doi: 10.1038/s41467-023-39424-2 (PMC10282047; doi:10.1038/s41467-023-39424-2)
Supplement: Supplementary file 1 — Supplementary Information [file 41467_2023_39424_MOESM1_ESM.pdf]

**SUPPLEMENTARY INFORMATION**

# CAJAL enables analysis and integration of single-cell morphological data using metric geometry

Kiya W. Govek[1], Patrick Nicodemus[1], Yuxuan Lin[2], Jake Crawford[3], Artur B. Saturnino[2], Hannah Cui[2], Kristi Zoga[1], Michael P. Hart[1], and Pablo G. Camara[1,4,5]

[1] Department of Genetics, Perelman School of Medicine, University of Pennsylvania, Philadelphia, PA 19104.

[2] Department of Mathematics, School of Arts and Sciences, University of Pennsylvania, Philadelphia, PA 19104

[3] Genomics and Computational Biology Graduate Group, Perelman School of Medicine, University of Pennsylvania, Philadelphia, PA 19104

[4] Institute for Biomedical Informatics, Perelman School of Medicine, University of Pennsylvania, Philadelphia, PA 19104

[5] Center for Artificial Intelligence and Data Science for Integrated Diagnostics, Perelman School of Medicine, University of Pennsylvania, Philadelphia, PA 19104.

Correspondence should be addressed to P.G.C. (email: pcamara@pennmedicine.upenn.edu)

## Supplementary Figures

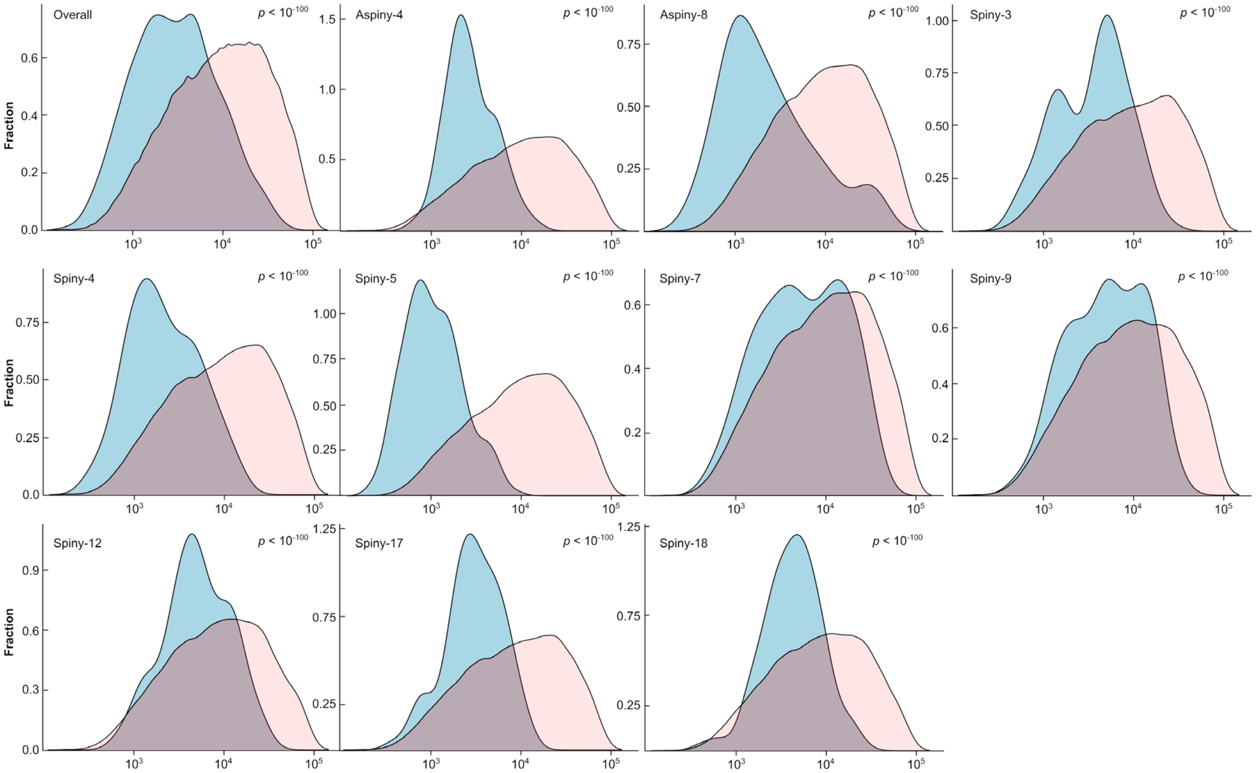


**Supplementary Figure 1. Consistency of previously reported neuronal m-types in the cell morphology space produced with CAJAL.** The distributions of pairwise GW distances between cells of the same m-type (blue) and between cells of different m-type (red) are shown for the morphology space of the basal and apical dendrites of visual cortex neurons profiled with patch-clamp[1]. In addition to the overall distribution, the distributions restricted to cells from each major m-type are shown. m-types were defined in Gouwens et al.[1] by hierarchical clustering using lists of neuronal morphological features. The two-sided Wilcoxon rank-sum test *p*-value is indicated in each case.

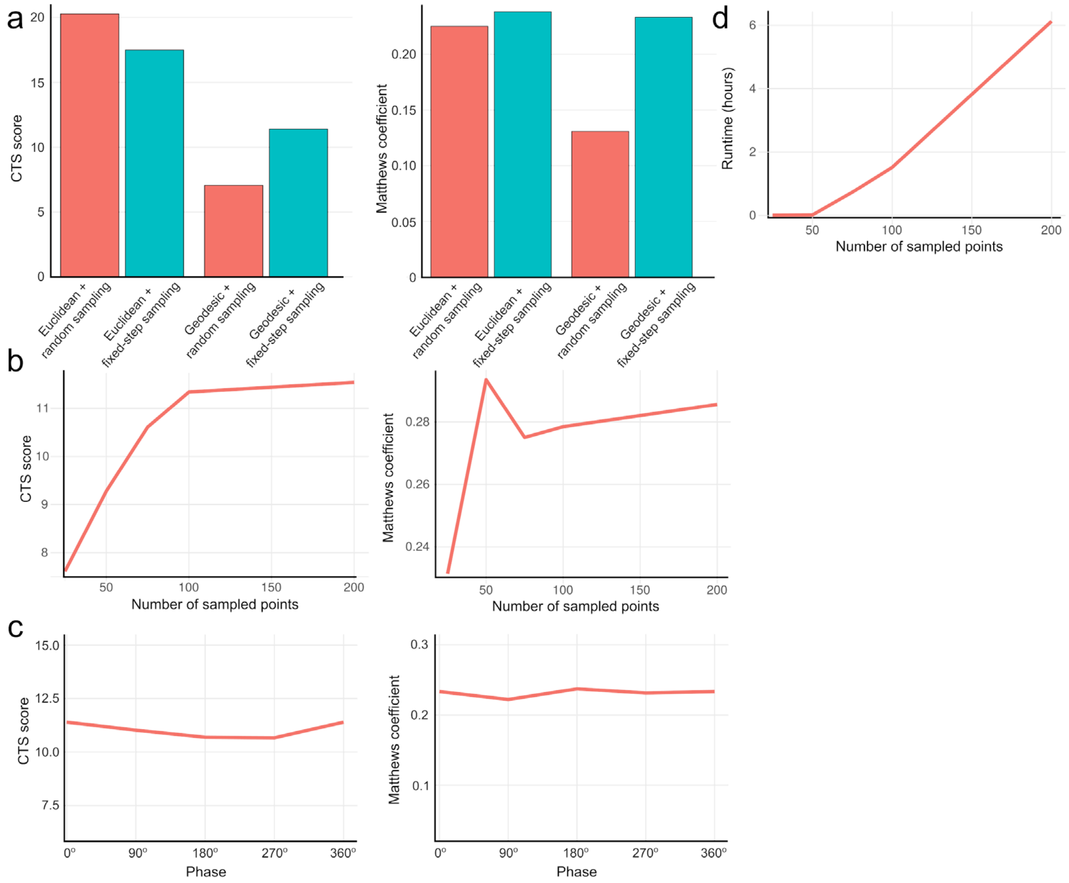


**Supplementary Figure 2. Performance of CAJAL as a function of sampling approach a distance metric. a)** The cell-type separation (CTS) score and the Matthews coefficient of a $k = 10$ nearest neighbor classifier were used to evaluate the ability of CAJAL to capture morphological differences between visual cortex neurons labelled with different Cre driver lines[1] for different choices of the approach used to sample points from each cell (random uniform sampling or fixed-step sampling) and the metric used to meassure distances between the sampled points (geodesic or Euclidean distance). **b-c)** The same metrics of performance as a function of the number of sampled points (b) and the initial sampled point (c), for fixed-step sampling and geodesic distance. **d)** Runtime as a function of the number of sampled points, for fixed-step sampling and geodesic distance. Runtime was determined based on 12 threads of a desktop computer with an 8-core Intel Xeon E5-1660 3.20 GHz CPU. Source data are provided as a Source Data file.

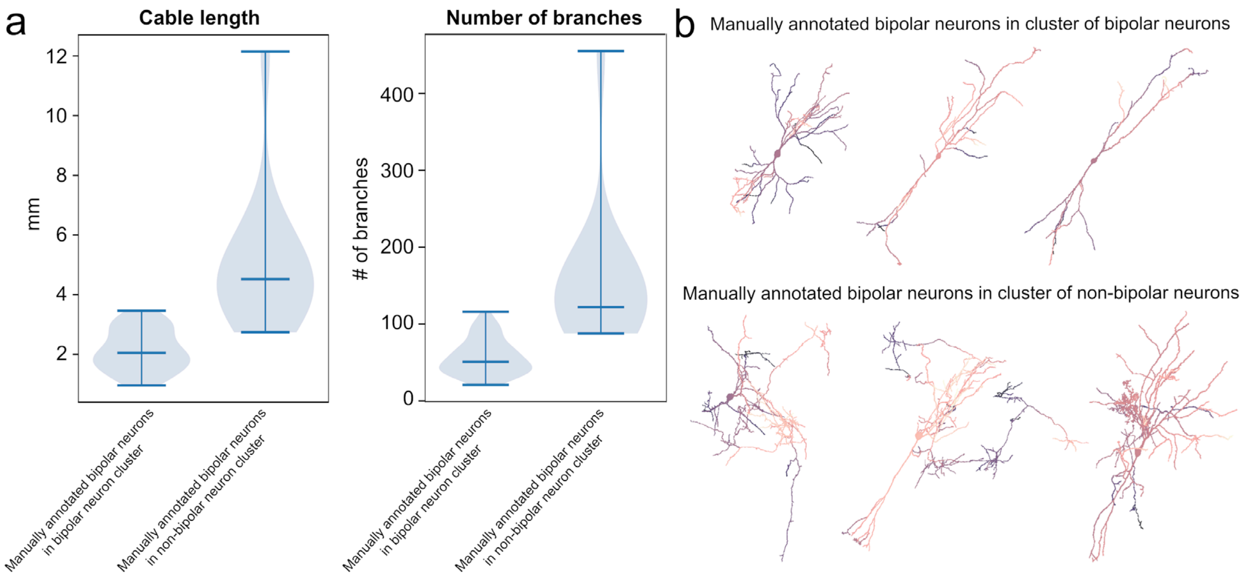


**Supplementary Figure 3. Classification of manually annotated bipolar neurons from the MICrONS Consortium dataset. a)** Cells that were annotated as bipolar neurons by the MICrONS Consortium[2] and classified as non-bipolar neurons by CAJAL have larger morphological complexity than correctly classified bipolar neurons. **b)** Representative examples of cells that were annotated as bipolar neurons by the MICrONS Consortium and classified as bipolar (top) or non-bipolar (bottom) neurons by CAJAL. The digital reconstructions of bipolar neurons that CAJAL misclassified as non-bipolar neurons have a more complex morphology, likely due to segmentation artifacts. Source data are provided as a Source Data file.

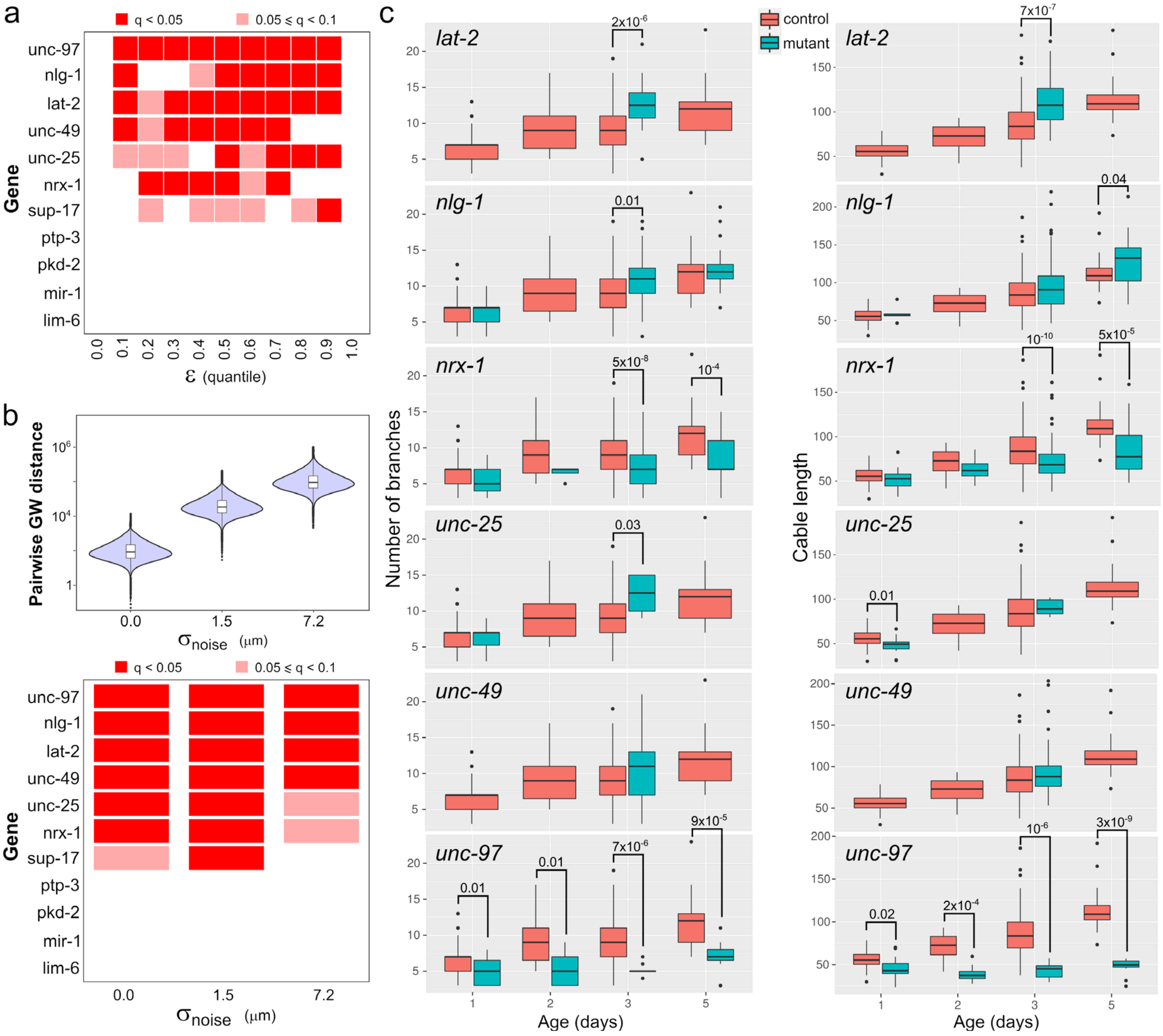


**Supplementary Figure 4. Identification of mutations that have an impact on the morphology of the DVB neuron. a)** Significant genes as a function of the radius parameter (ε) of the Laplacian score expressed as quantiles of the distribution of GW distances and for two levels of significance (FDR < 0.05 and FDR < 0.1). The set of genes that are found to significantly affect the morphology of the DVB neuron is consistent across different choices of the radius parameter. **b)** Significant genes as a function of the amount of noise added to the neuronal digital reconstructions. The coordinates of each point in the digital reconstructions of the DVB neuron were shifted by random amounts sampled from a Gaussian distribution with

mean 0 and standard deviation $\sigma_{\text{noise}}$ corresponding to 0%, 10%, and 50% of the total standard deviation of the coordinates. Top: distribution of GW pairwise distances between cells as a function of the amount of added noise (*n* = 318,801 pairwise distances per condition). The boxplots denote the median (line), interquartile range (IQR) (box), min(max(x), Q3+1.5 IQR) (upper whisker), and max(min(x), Q1-1.5 IQR) (lower whisker), with Q1 and Q3 the first and third quartiles. Bottom: significant genes as a function of the amount of added noise and for two levels of significance (FDR < 0.05 and FDR < 0.1). **c)** Number of branches and cable length of the DVB neuron in control and mutant worms (day 1: *n* = 47 control worms, 57 mutant worms; day 2: *n* = 11 control worms, 13 mutant worms; day 3: *n* = 195 control worms, 374 mutant worms; day 5: *n* = 25 control worms, 77 mutant worms). The number of branches and total cable length is shown for each of the 6 significantly associated genes with the morphology of the DVB neuron that were identified with CAJAL. The boxplots denote the median (line), interquartile range (IQR) (box), min(max(x), Q3+1.5 IQR) (upper whisker), and max(min(x), Q1-1.5 IQR) (lower whisker), with Q1 and Q3 the first and third quartiles. Source data are provided as a Source Data file.

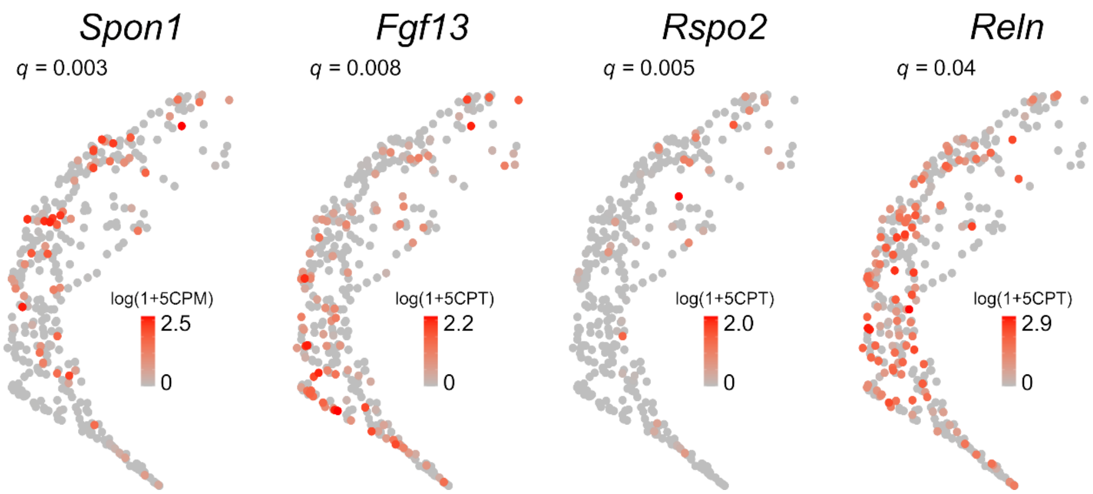


**Supplementary Figure 5. Secreted factors associated with morpho-transcriptomic trajectories of inhibitory neurons.** The UMAP representation of the cell morphology space of the dendrites of 370 inhibitory neurons is colored by the gene expression level of 4 genes coding for secreted factors that are significantly associated with both the RNA velocity field and the structure of the cell morphology space (Laplacian score permutation test, FDR < 0.05). The *q*-value of the Laplacian score is shown for each gene. CPT: counts per thousand.

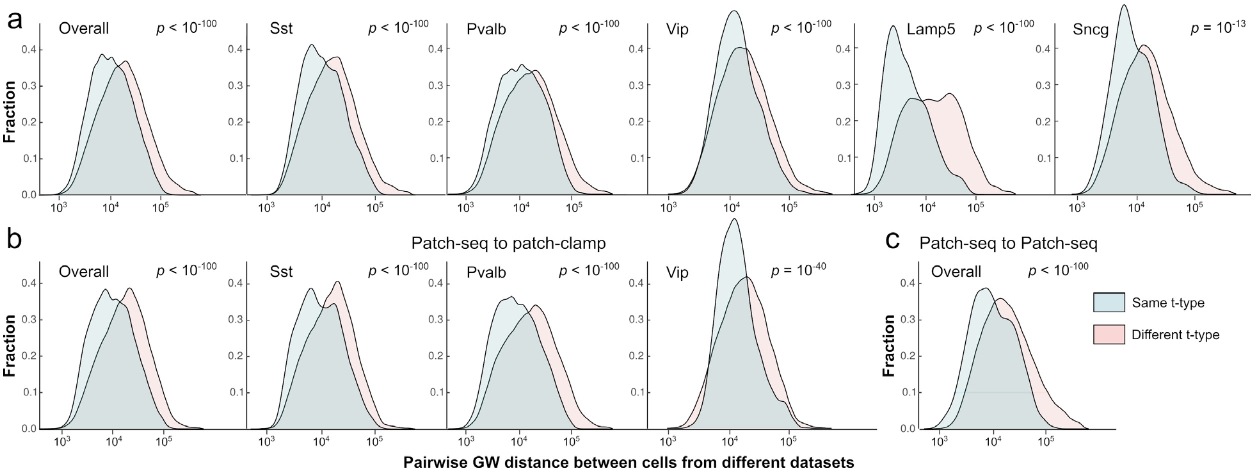


**Supplementary Figure 6. Consistency of major t-types across datasets in the integrated cell morphology space of Patch-seq and patch-clamp datasets. a)** The distributions of pairwise GW distances between cells of the same transcriptomic class (blue) and between cells of different transcriptomic class (red), where each cell in the pair belongs to a different dataset, are shown for the combined morphology space of the basal and apical dendrites of visual cortex neurons profiled with patch-clamp[1], and visual cortex and motor cortex neurons profiled with Patch-seq[3,4]. In addition to the overall distributions, the distributions restricted to cells from each major transcriptomic class are shown. The two-sided Wilcoxon rank-sum test *p*-value is indicated in each case. **b)** Same as in (a) but restricting to only distances between Patch-seq and patch-clamp cells. **c)** Same as in (a) but restricting to only distances between cells from the two Patch-seq datasets.

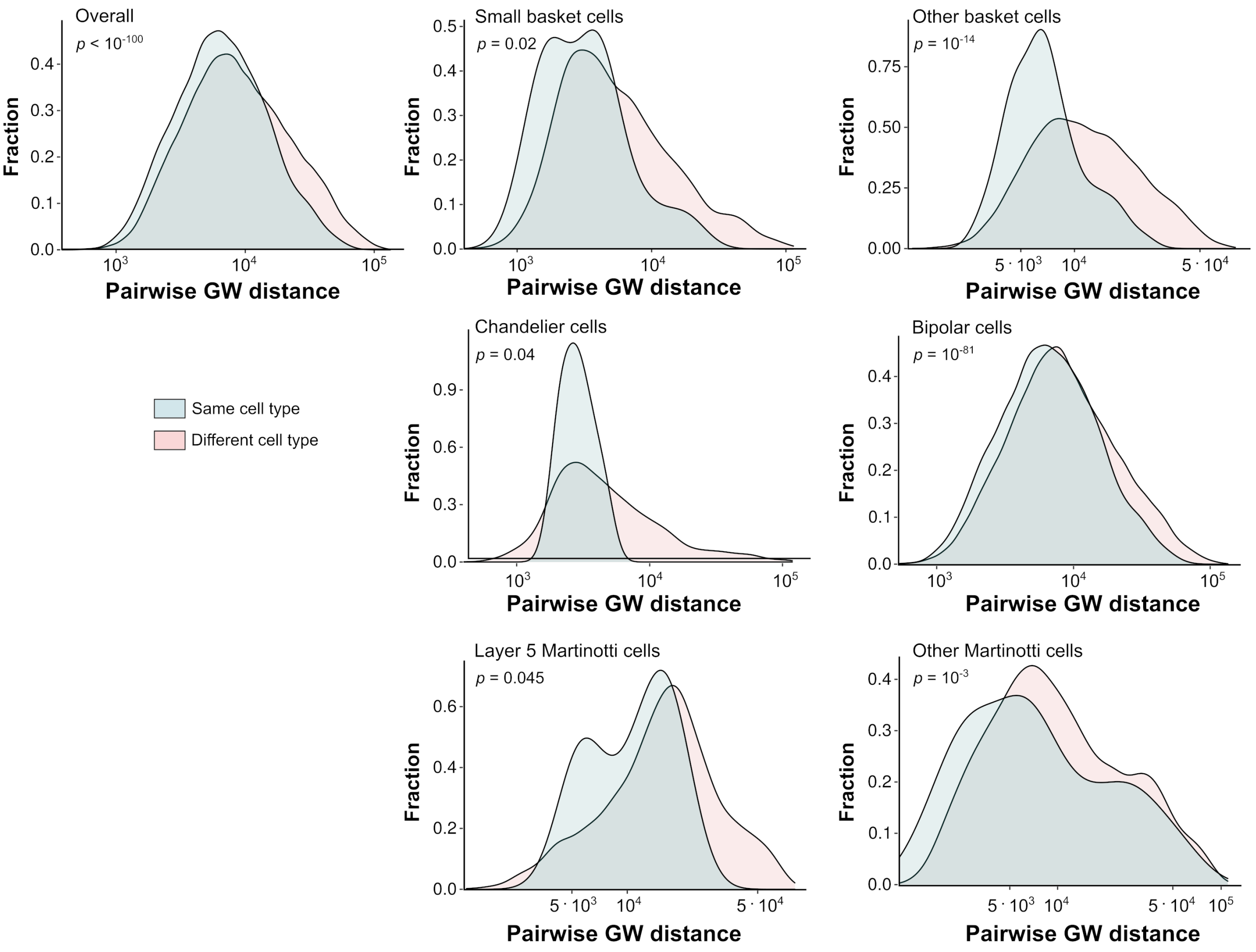


**Supplementary Figure 7. Consistency of cell types across datasets in the integrated cell morphology space of Patch-seq and MICrONS datasets.** The distributions of pairwise GW distances between cells of the same type (blue) and between cells of different type (red), where one cell in the pair belongs to the MICrONS dataset and the other to one of the Patch-seq datasets, are shown for the full reconstructions visual cortex and motor cortex neurons profiled with Patch-seq[3,4] and visual cortical neurons profiled with serial electron microscopy by the MICrONS program[2]. Cell types in the Patch-seq datasets were annotated based on their t-type. In addition to the overall distributions, the distributions restricted to cells from each type are shown. The two-sided Wilcoxon rank-sum test *p*-value is indicated in each case.

## Supplementary Tables

| | Gouwens et al. 2019 **(Patch-clamp)** | | Scala et al. 2020 **(Patch-seq)** | | Gouwens et al. 2020 **(Patch-seq)** | | Peng et al. 2021 **(fMOST)** | | Avg. CTS score rel. to CAJAL |
|---|---|---|---|---|---|---|---|---|---|
| | *CTS score* | *CTS score rel. to CAJAL* | *CTS score* | *CTS score rel. to CAJAL* | *CTS score* | *CTS score rel. to CAJAL* | *CTS score* | *CTS score rel. to CAJAL* | |
| NBLAST | 1.06 | 6.1% | 1.04 | 6.5% | 1.03 | 29.4% | 1 | 15.1% | 14.3% |
| TMD | 2.59 | 14.8% | 2.69 | 16.8% | 2.17 | 62.0% | 2.63 | 39.8% | 33.3% |
| L-Measure | 6.19 | 35.4% | 3.05 | 19.0% | 1.36 | 38.9% | 6.06 | 91.8% | 46.3% |
| SNT | 2.21 | 12.6% | 2.21 | 13.8% | 1.09 | 31.1% | 4.32 | 65.4% | 30.7% |
| Sholl | 2.12 | 12.1% | 1.6 | 10.0% | 1.13 | 32.3% | 1.84 | 27.9% | 20.6% |
| ElasticP2P | 14.97 | 85.5% | 2.15 | 13.4% | 1.68 | 48.0% | 2.95 | 44.7% | 47.9% |
| **CAJAL** | 17.5 | 100% | 16.05 | 100% | 3.5 | 100% | 6.6 | 100% | 100% |

**Supplementary Table 1. Cell-type separation (CTS) score of CAJAL and six other methods for neuronal morphometry in the identification of morphological differences between molecularly and anatomically defined neurons across four datasets.**

| | Gouwens et al. 2019 **(Patch-clamp)** | | | | Scala et al. 2020 **(Patch-seq)** | | | | Gouwens et al. 2020 **(Patch-seq)** | | | | Peng et al. 2021 **(fMOST)** | | | | Avg. accuracy relative to CAJAL (comb. p-value) | Avg. MCC relative to CAJAL (comb. p-value) |
|---|---|---|---|---|---|---|---|---|---|---|---|---|---|---|---|---|---|---|
| | *Acc.* | *Acc. rel. to CAJAL (p-val.)* | *MCC* | *MCC rel. to CAJAL (p-val.)* | *Acc.* | *Acc. rel. to CAJAL (p-val.)* | *MCC* | *MCC rel. to CAJAL (p-val.)* | *Acc.* | *Acc. rel. to CAJAL (p-val.)* | *MCC* | *MCC rel. to CAJAL (p-val.)* | *Acc.* | *Acc. rel. to CAJAL (p-val.)* | *MCC* | *MCC rel. to CAJAL (p-val.)* | | |
| NBLAST | 16.5% | 56.2% ($6\cdot10^{-6}$) | 0.10 | 42.2% ($6\cdot10^{-6}$) | 29.9% | 58.1% ($9\cdot10^{-5}$) | 0.18 | 43.1% ($6\cdot10^{-6}$) | 56.6% | 88.5% ($9\cdot10^{-5}$) | 0.34 | 77.1% ($6\cdot10^{-6}$) | 33.8% | 51.3% ($9\cdot10^{-5}$) | 0.1 | 17.4% ($6\cdot10^{-6}$) | 63.6% ($5\cdot10^{-14}$) | 44.9% ($3\cdot10^{-17}$) |
| TMD | 18.9% | 64.2% ($6\cdot10^{-6}$) | 0.12 | 51.9% ($6\cdot10^{-6}$) | 53.8% | 104.5% ($3\cdot10^{-4}$) | 0.44 | 106.6% ($2\cdot10^{-4}$) | 63.4% | 99.2% (0.09) | 0.43 | 98.4% (0.14) | 40.9% | 62.2% ($6\cdot10^{-6}$) | 0.26 | 45.4% ($6\cdot10^{-6}$) | 82.5% ($10^{-8}$) | 75.6% ($2\cdot10^{-8}$) |
| L-Measure | 15.1% | 51.3% ($9\cdot10^{-5}$) | 0.08 | 35.6% ($6\cdot10^{-6}$) | 36.2% | 70.3% ($9\cdot10^{-5}$) | 0.22 | 54.3% ($6\cdot10^{-6}$) | 51.8% | 81.1% ($6\cdot10^{-6}$) | 0.21 | 48.5% ($6\cdot10^{-6}$) | 46.4% | 70.6% ($6\cdot10^{-6}$) | 0.32 | 55.00% ($6\cdot10^{-6}$) | 68.3% ($4\cdot10^{-15}$) | 48.3% ($3\cdot10^{-17}$) |
| SNT | 22.9% | 77.9% ($6\cdot10^{-6}$) | 0.17 | 70.7% ($6\cdot10^{-6}$) | 44.8% | 86.9% ($9\cdot10^{-5}$) | 0.32 | 78.8% ($6\cdot10^{-6}$) | 57.3% | 89.6% ($6\cdot10^{-6}$) | 0.31 | 70.5% ($6\cdot10^{-6}$) | 57.9% | 88.1% ($6\cdot10^{-6}$) | 0.47 | 82.4% ($6\cdot10^{-6}$) | 85.6% ($3\cdot10^{-16}$) | 75.6% ($3\cdot10^{-17}$) |
| Sholl | 19.0% | 64.9% ($9\cdot10^{-5}$) | 0.13 | 53.1% ($6\cdot10^{-6}$) | 37.2% | 72.2% ($9\cdot10^{-5}$) | 0.23 | 55.7% ($6\cdot10^{-6}$) | 51.8% | 81.0% ($9\cdot10^{-5}$) | 0.21 | 47.2% ($6\cdot10^{-6}$) | 28.9% | 43.9% ($6\cdot10^{-6}$) | 0.09 | 15.8% ($6\cdot10^{-6}$) | 65.5% ($5\cdot10^{-14}$) | 43.0% ($3\cdot10^{-17}$) |
| ElasticP2P | 9.5% | 32.4% ($9\cdot10^{-5}$) | 0.04 | 18.0% ($6\cdot10^{-6}$) | 44.1% | 85.6% ($9\cdot10^{-5}$) | 0.32 | 77.1% ($6\cdot10^{-6}$) | 46.8% | 73.2% ($9\cdot10^{-5}$) | 0.22 | 49.4% ($6\cdot10^{-6}$) | 24.7% | 37.6% ($6\cdot10^{-6}$) | 0.07 | 11.5% ($6\cdot10^{-6}$) | 57.2% ($5\cdot10^{-14}$) | 39.0% ($3\cdot10^{-17}$) |
| Random | 5.4% | 18.3% ($9\cdot10^{-5}$) | 0 | 0% | 16.9% | 32.8% ($9\cdot10^{-5}$) | 0 | 0% | 31.9% | 49.9% ($9\cdot10^{-5}$) | 0 | 0% | 20.3% | 30.8% ($9\cdot10^{-5}$) | 0 | 0% | 32.9% ($6\cdot10^{-13}$) | 0% |
| **CAJAL** | 29.4% | 100% | 0.24 | 100% | 51.5% | 100% | 0.41 | 100% | 63.9% | 100% | 0.44 | 100% | 65.8% | 100% | 0.57 | 100% | 100% | 100% |

**Supplementary Table 2. Accuracy and Matthews Correlation Coefficient of CAJAL and six other methods for neuronal morphometry in the prediction of the molecular type of neurons in four datasets.** Average values and statistical significance were estimated using 10 random initializations of a 7-fold nearest-neighbors classifier. One-tailed Wilcoxon rank-sum test. Combined *p*-values were obtained using Fisher's method. Acc.: Accuracy; rel.: relative; MCC: Matthews Correlation Coefficient; Avg.: Average; p-val: p-value; comb.: combined. Source data are provided as a Source Data file.

| Mutant background | Array name | *n* (day 1) | *n* (day 2) | *n* (day 3) | *n* (day 5) |
|---|---|---|---|---|---|
| *him-5(e1490)* | *otIs541* | 34 | 5 | 116 | 4 |
| *him-8(e1489)* | *otIs525* | 13 | 6 | 79 | 21 |
| *lim-6(nr2073); him-5(e1490)* | *otIs541* | 0 | 0 | 9 | 0 |
| *unc-97(su110); him-5(e1490)* | *otIs541* | 14 | 9 | 9 | 11 |
| *nlg-1(ok259); him-5(e1490)* | *otIs541* | 5 | 0 | 34 | 29 |
| *unc-119(ed3); nrx-1(wy778[unc-119(+)]); him-8(e1489)* | *otIs525* | 11 | 4 | 77 | 37 |
| *unc-49(e407); him-5(e1490)* | *otIs541* | 0 | 0 | 43 | 0 |
| *pkd-2(sy606); him-5(e1490)* | *otIs541* | 0 | 0 | 9 | 0 |
| *unc-25(e156); him-5(e1490)* | *otIs541* | 18 | 0 | 6 | 0 |
| *mir-1(n4102); him-8(e1489)* | *otIs541* | 5 | 0 | 34 | 0 |
| *lat-2(tm463); him-5(e1490)* | *otIs525* | 0 | 0 | 24 | 0 |
| *sup-17(n1258); him-8(e1489)* | *otIs541* | 0 | 0 | 28 | 0 |
| *ptp-3(ok244); him-5(e1490)* | *otIs525* | 0 | 0 | 22 | 0 |
| *nlg-1(ok259); lat-2(tm463); him-5(e1490)* | *otis525* | 0 | 0 | 8 | 0 |
| *unc-119(ed3); nrx-1(wy778[unc-119(+)]); mir-1(n4102); him-8(e1489)* | *otis525* | 0 | 0 | 6 | 0 |
| *nlg-1(ok259); unc-119(ed3); nrx-1(wy778[unc-119(+)]); him-8(e1489)* | *otis525* | 0 | 0 | 43 | 0 |
| *mir-1(n4102); nlg-1(ok259); him-8(e1489)* | *otis541* | 0 | 0 | 13 | 0 |
| *unc-49(e407); nlg-1(ok259); him-5(e1490)* | *otis541* | 0 | 0 | 9 | 0 |
| *unc-119(ed3); nrx-1(wy778[unc-119(+)]); him-8(e1489)* | *otis541* | 4 | 0 | 0 | 0 |

**Supplementary Table 3. *C. elegans* strains used in the morphological analysis of the DVB neuron.**

**Supplementary References**


1 Gouwens, N. W. *et al.* Classification of electrophysiological and morphological neuron types in the mouse visual cortex. *Nat Neurosci* **22**, 1182-1195, doi:10.1038/s41593-019-0417-0 (2019).

2 MICrONS Consortium *et al.* Functional connectomics spanning multiple areas of mouse visual cortex. *bioRxiv*, 2021.2007.2028.454025, doi:10.1101/2021.07.28.454025 (2021).

3 Gouwens, N. W. *et al.* Integrated Morphoelectric and Transcriptomic Classification of Cortical GABAergic Cells. *Cell* **183**, 935-953 e919, doi:10.1016/j.cell.2020.09.057 (2020).

4 Scala, F. *et al.* Phenotypic variation of transcriptomic cell types in mouse motor cortex. *Nature* **598**, 144-150, doi:10.1038/s41586-020-2907-3 (2021).
