# Supplementary material for: CAJAL enables analysis and integration of single-cell morphological data using metric geometry
Source: Nat Commun. 2023 Jun 21;14:3672. doi: 10.1038/s41467-023-39424-2 (PMC10282047; doi:10.1038/s41467-023-39424-2)
Supplement: Supplementary file 3 — Description of additional supplementary files [file 41467_2023_39424_MOESM3_ESM.pdf]

Description of additional supplementary files

Supplementary Dataset 1. Morphological features associated with the structure of the cell morphology space of the DVB neuron. The Laplacian score, p-value, and q-value are presented for each of 33 morphological features evaluated in the cell morphology space of the DVB neuron. One-sided permutation test, FDR controlled using Benjamini-Hochberg procedure.

Supplementary Dataset 2. Genes associated with the structure of the cell morphology space of the basal and apical dendrites motor cortex neurons profiled with Patch-seq. The Laplacian score, p-value, and q-value are presented for the expression of each gene evaluated in the cell morphology spaces of excitatory and inhibitory neurons. The column “t-type associated” indicates whether the gene is also significantly associated with one or several ttypes (FDR < 0.1). One-sided permutation test, FDR controlled using Benjamini-Hochberg procedure.

Supplementary Dataset 3. Electrophysiological features associated with the structure of the cell morphology space of the basal and apical dendrites motor cortex neurons profiled with Patch-seq. The Laplacian score, p-value, and q-value are presented for each of 29 electrophysiological features evaluated in the cell morphology spaces of excitatory and inhibitory neurons. One-sided permutation test, FDR controlled using Benjamini-Hochberg procedure.

Supplementary Dataset 4. Genes associated with morpho-transcriptomic trajectories of inhibitory motor cortex neurons profiled with Patch-seq. The Laplacian score, p-value, and q-value evaluated in the cell morphology space are presented for each of the 78 genes associated with the RNA velocity field. One-sided permutation test, FDR controlled using Benjamini-Hochberg procedure
