# Supplementary material for: CAJAL enables analysis and integration of single-cell morphological data using metric geometry
Source: Nat Commun. 2023 Jun 21;14:3672. doi: 10.1038/s41467-023-39424-2 (PMC10282047; doi:10.1038/s41467-023-39424-2)
Supplement: Supplementary file 8 — Reporting Summary [file 41467_2023_39424_MOESM8_ESM.pdf]

nature portfolio

Corresponding author(s): Pablo G. Camara

Last updated by author(s): Jun 5, 2023

Reporting Summary

Nature Portfolio wishes to improve the reproducibility of the work that we publish. This form provides structure for consistency and transparency in reporting. For further information on Nature Portfolio policies, see our Editorial Policies and the Editorial Policy Checklist.

Statistics

For all statistical analyses, confirm that the following items are present in the figure legend, table legend, main text, or Methods section.

n/a	Confirmed	
☐	☒	The exact sample size (*n*) for each experimental group/condition, given as a discrete number and unit of measurement
☐	☒	A statement on whether measurements were taken from distinct samples or whether the same sample was measured repeatedly
☐	☒	The statistical test(s) used AND whether they are one- or two-sided<br>*Only common tests should be described solely by name; describe more complex techniques in the Methods section.*
☐	☒	A description of all covariates tested
☐	☒	A description of any assumptions or corrections, such as tests of normality and adjustment for multiple comparisons
☐	☒	A full description of the statistical parameters including central tendency (e.g. means) or other basic estimates (e.g. regression coefficient) AND variation (e.g. standard deviation) or associated estimates of uncertainty (e.g. confidence intervals)
☐	☒	For null hypothesis testing, the test statistic (e.g. *F*, *t*, *r*) with confidence intervals, effect sizes, degrees of freedom and *P* value noted<br>*Give P values as exact values whenever suitable.*
☒	☐	For Bayesian analysis, information on the choice of priors and Markov chain Monte Carlo settings
☒	☐	For hierarchical and complex designs, identification of the appropriate level for tests and full reporting of outcomes
☐	☒	Estimates of effect sizes (e.g. Cohen's *d*, Pearson's *r*), indicating how they were calculated

Our web collection on statistics for biologists contains articles on many of the points above.

Software and code

Policy information about availability of computer code

Data collection	Simple Neurite Tracer (SNT) 3.2.12
Data analysis	CAJAL (https://github.com/CamaraLab/CAJAL and https://cajal.readthedocs.io), python 3.7.3 and 3.10.5, R 3.4.1, MATLAB R2022b, networkx 2.4, umap 0.2.0.1 (https://github.com/tkonopka/umap), igraph 1.2.6, umap-learn 0.5.3, leidenalg 0.8.10, ot 0.7.0, RayleighSelection (https://github.com/CamaraLab/RayleighSelection), scikit-learn 1.0.2, nat.nblast 1.6.6, tmd 2.0.12, ImageJ/Fiji 1.53c, SNT 3.2.12, L measure (Lmv) 5.3, fpc 2.1-11, meshparty 1.16.4, caveclient 4.5.0, potpourri3d 0.0.7, numpy 1.22.4, hdbscan 0.8.28, Celltool 2.0, Cellprofiler 4.0.3, SPHARM 1.0, gProfileR 0.6.7, scvelo 0.2.4, velocyto 0.17.13, STAR 2.7.1

For manuscripts utilizing custom algorithms or software that are central to the research but not yet described in published literature, software must be made available to editors and reviewers. We strongly encourage code deposition in a community repository (e.g. GitHub). See the Nature Portfolio guidelines for submitting code & software for further information.

Data

Policy information about availability of data

All manuscripts must include a data availability statement. This statement should provide the following information, where applicable:

- Accession codes, unique identifiers, or web links for publicly available datasets
- A description of any restrictions on data availability
- For clinical datasets or third party data, please ensure that the statement adheres to our policy

All the datasets used in this study are publicly available. The morphological reconstructions of the DVB neuron generated in this study have been deposited in the neuromorpho.org database (Hart archive). The patch-clamp data of Gouwens et al.34 are available at the Allen Brain Atlas data portal (http://celltypes.brain-map.org/data). The Patch-seq datasets of Gouwens et al.36 and Scala et al.37 are available at the Brain Image Library (BIL) using the URLs https://download.brainimagelibrary.org/biccn/zeng/pseq/morph/200526/ and https://download.brainimagelibrary.org/biccn/zeng/tolias/pseq/morph/, respectively. The fMOST dataset of Peng et al.38 are available at the BIL using the URL https://download.brainimagelibrary.org/biccn/zeng/luo/fMOST/cells/. The two-photon microscopy data of Medyukhina et al.21 are available at https://asbdata.hki-jena.de/publidata/MedyukhinaEtAL_SPHARM/. The MICrONS program dataset is available at the MICrONS Explorer (https://www.microns-explorer.org/cortical-mm3#segmentation-meshes). The GRCm38 mouse reference genome is available at https://www.ncbi.nlm.nih.gov/datasets/genome/GCF_000001635.20/. Source data are provided with this paper.

Human research participants

Policy information about studies involving human research participants and Sex and Gender in Research.

Reporting on sex and gender	N/A
Population characteristics	N/A
Recruitment	N/A
Ethics oversight	N/A

Note that full information on the approval of the study protocol must also be provided in the manuscript.

Field-specific reporting

Please select the one below that is the best fit for your research. If you are not sure, read the appropriate sections before making your selection.

☒ Life sciences ☐ Behavioural & social sciences ☐ Ecological, evolutionary & environmental sciences

For a reference copy of the document with all sections, see nature.com/documents/nr-reporting-summary-flat.pdf

Life sciences study design

All studies must disclose on these points even when the disclosure is negative.

Sample size	We considered all the available samples from the Patch-seq, intra vital two-photon microscopy, and MICrONS datasets used in this study, consisting of 509 neurons (Gouwens et al. 2019), 574 neurons (Gouwens et al. 2020), 645 neurons (Scala et al. 2021), 512 T cells (Medyukhina et al. 2020), and 113,182 brain cells (MICrONS Consortium et al. 2021). These sample sizes were chosen based on sample availability, correspond to some of the largest single-cell morphological datasets that are currently available, and led to statistically significant and reproducible results in our analyses. For the DVB neuron morphology data, sample size was uniformly determined by analyzing at least 3 animals for each condition, and more than 10 animals in most cases.
Data exclusions	We did not process the SWC files of 3 neurons from the (Gouwens et al. 2019) dataset since they were not sorted. 62 neurons from the (Gouwens et al. 2020) dataset did not have assigned transcriptomic type and were therefore not considered in the analyses. The dendrites of one inhibitory neuron from the (Scala et al. 2021) dataset were not present and this neuron was therefore not considered in the analyses. 42,672 meshes from the MICrONS dataset were identified as artifacts or doublets after the filtering and quality control process detailed in the Methods section of the manuscript, and were therefore not considered in downstream analyses.
Replication	Confocal imaging of control and mutant worms was performed across multiple days and replicates with three independent replicates for each genotype. We verified in all analyses that the results were stable against different choices of the clustering and visualization parameters.
Randomization	The order and allocation of controls and mutants was randomized for each replicate.
Blinding	Blinding to group (worm genotype) allocation is not relevant to this study, since all statistical tests involve the comparison between mutants and controls.

Reporting for specific materials, systems and methods

We require information from authors about some types of materials, experimental systems and methods used in many studies. Here, indicate whether each material, system or method listed is relevant to your study. If you are not sure if a list item applies to your research, read the appropriate section before selecting a response.

Materials & experimental systems

n/a	Involved in the study
☒	☐ Antibodies
☒	☐ Eukaryotic cell lines
☒	☐ Palaeontology and archaeology
☒	☐ Animals and other organisms
☒	☐ Clinical data
☒	☐ Dual use research of concern

Methods

n/a	Involved in the study
☒	☐ ChIP-seq
☒	☐ Flow cytometry
☒	☐ MRI-based neuroimaging
